# Genetic Diversity and Forensic Utility of X-STR Loci in Punjabi and Kashmiri Populations: Insights into Population Structure and Ancestry

**DOI:** 10.3390/genes15111384

**Published:** 2024-10-28

**Authors:** Muhammad Farhan Khan, Allah Rakha, Anam Munawar, Shahid Nazir, Arman Khan, Muhammad Adnan Khan, Munir Ahmad, Chuan-Chao Wang, Atif Adnan

**Affiliations:** 1 Department of Forensic Medicine, University of Health Sciences, Lahore 54600, Pakistan; 2 School of Computing, Skyline University College, University City Sharjah, Sharjah 1797, United Arab Emirates; 3 Riphah School of Computing & Innovation, Faculty of Computing, Riphah International University, Lahore Campus, Lahore 54000, Pakistan; 4 Department of Software, Faculty of Artificial Intelligence and Software, Gachon University, Seongnam-si 13120, Republic of Korea; 5 Department of Computer Science, National College of Business Administration and Economics, Lahore 54009, Pakistan; 6 College of Informatics, Korea University, Seoul 02841, Republic of Korea; 7 Department of Anthropology and Ethnology, Institute of Anthropology, School of Sociology and Anthropology, Xiamen University, Xiamen 361000, China

**Keywords:** X-chromosomal STRs, forensic genetics, population structure, Punjabi and Kashmiri populations, genetic ancestry analysis

## Abstract

**Background:** X-chromosomal short tandem repeats (X-STRs) are crucial in forensic applications, particularly in complex kinship cases, and play an important role in population genetics. However, there is limited data on X-STR variation in Pakistani populations, especially among ethnic groups like Kashmiri and Punjabi. **Methodology:** This study investigates the forensic and genetic properties of 12 X-STRs from the Investigator Argus X-12 Kit (QIAGEN, Hilden, Germany) in 125 families (75 Kashmiri, 50 Punjabi) from Azad Jammu and Kashmir and Punjab, Pakistan. **Results:** In both populations, a total of 222 alleles were identified across the 12 X-STR loci (Punjabi 171 alleles, Kashmiri 161 alleles), with allele frequencies ranging from 0.0056 to 0.3033. DXS10148 was the most polymorphic locus with 28 alleles, while DXS7132 was the least polymorphic with 9 alleles. Most loci were in linkage equilibrium, except for the DXS10135/DXS10148 pair in males, with no loci exhibiting significant linkage disequilibrium in females. The combined power of discrimination was 0.999 999 9977 for Kashmiri males, 0.999 999 999 999 9746 for Kashmiri females, and 0.999 999 999 999 9781 for Punjabi females. In Kashmiri males, 34, 31, 28, and 32 haplotypes were observed across the four linkage groups (LG1, LG2, LG3, and LG4), though these groups did not form stable haplotypes, as indicated by Linkage Equilibrium within and significant Linkage Disequilibrium between groups. **Conclusions:** Genetic structure analysis using Principal Component Analysis and STRUCTURE revealed distinct clustering patterns for the Kashmiri and Punjabi populations, indicating unique genetic backgrounds and ancestry influences, particularly distinguishing them from East Asian populations. This study provides a comprehensive analysis of X-STR variation in Punjabi and Kashmiri populations, offering valuable insights for forensic and population genetic studies.

## 1. Introduction

The Kashmiri and Punjabi populations, each with deep-rooted historical and cultural legacies, are two distinct but significant communities in South Asia. The Kashmiri people trace their ancestry to the sage Kashyapa, and their culture has evolved through centuries of Buddhist, Hindu, and Islamic influence, culminating in the philosophy of Kashmiriyat, which promotes unity and communal coexistence. The Kashmiri language, part of the Dardic branch of the Indo-Aryan family, has been shaped by Sanskrit, Persian, and Central Asian linguistic influences. Kashmiri traditions are further enriched by their renowned craftsmanship, cuisine, and diverse cultural heritage [1,2,3]. On the other hand, the Punjabi population, primarily located in the fertile Punjab region, has been shaped by a series of civilizations, including the Indus Valley, Persian, and Mughal empires. The Punjabi language, a cornerstone of regional identity, is written in Gurmukhi in India and Shahmukhi in Pakistan, underscoring the area’s religious and linguistic diversity. Punjabis are widely celebrated for their lively cultural expressions in music, dance, and festivals, as well as their strong agricultural tradition, which has made Punjab the agricultural hub of both India and Pakistan. Despite historical events like the 1947 partition, both communities retain strong social bonds and continue to shape the cultural and social fabric of their regions [4,5,6,7,8].

In forensic genetics, short tandem repeat (STR) loci are widely used for paternity testing and individual identification [9,10,11,12,13,14]. X-chromosomal short tandem repeats (X-STRs) have gained prominence in forensic applications, particularly in complex kinship analyses. X-STRs combine characteristics of both autosomal and uniparental genetic markers, making them particularly useful in establishing mother–son relationships, sibling relationships between females who share the same biological father, and grandmother–granddaughter relationships [15,16,17,18,19,20]. Additionally, X-STRs are advantageous in paternity cases involving female children, as men pass the same X chromosome to all their daughters, ensuring shared alleles at every X-STR locus [21]. The Investigator Argus X-12 Kit (QIAGEN, Hilden, Germany) is one of the most widely used multiplex kits for X-STRs, including 12 X-STR markers and the amelogenin locus, and has been employed in numerous population studies worldwide [22,23,24,25].

Despite the established utility of X-STRs in forensic and genetic studies, data on certain populations in Pakistan, such as the Kashmiris and Punjabis, remain limited. This study aims to investigate the forensic and population genetic properties of 12 X-STRs using the Investigator Argus X-12 Kit in Punjabi and Kashmiri populations from Punjab and Azad Kashmir, Pakistan. The objective is to provide comprehensive data on allele frequencies, linkage equilibrium, and haplotype diversity, contributing to the broader understanding of genetic variation in these populations and enhancing the applicability of X-STR markers in forensic and population genetic studies in these regions.

## 2. Materials and Methods

### 2.1. Population Samples

**Figure 1:**
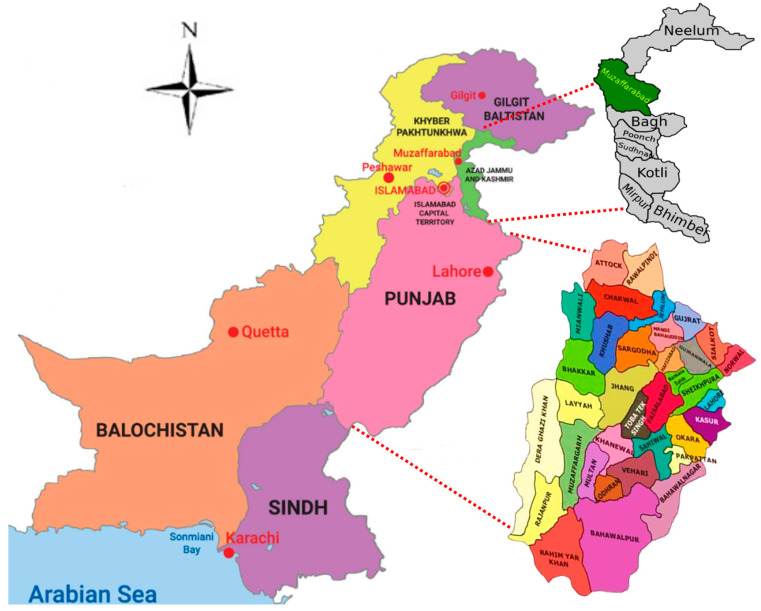
Map of Pakistan showing Punjab and Azad Jammu and Kashmir.

To investigate the forensic parameters and genetic structure of the Punjabi and Kashmiri populations from Punjab and Azad Jammu and Kashmir in Pakistan (Figure 1), blood samples were collected from 125 volunteer donors (75 Kashmiri, of which 35 males and 40 females and 50 Punjabi which were females) with informed consent. DNA was extracted using the Chelex-100 method [26] DNA concentration and purity were measured using a Nanodrop-2000 spectrophotometer (Thermo Fisher Scientific, Wilmington, DE, USA), and the DNA was stored at −20 °C until amplification.

### 2.2. Compliance with Ethics Guidelines

This study received approval from the ethical review board of the University of Health Sciences Lahore, Pakistan, adhering to the principles outlined in the Declaration of Helsinki.

### 2.3. PCR Amplification and STR Typing

Diluted DNA extracts and positive control samples (9947A and 9948) were amplified using the Investigator Argus X-12 Kit on a GeneAmp PCR System 9700 Thermal Cycler (Thermo Fisher Scientific, Waltham, MA, USA), following the manufacturer’s protocol. PCR products were analyzed with an 8-capillary ABI 3500 DNA Genetic Analyzer using POP-4™ polymer (Life Technologies, Carlsbad, CA, USA), according to the manufacturer’s instructions. Genotypes were assigned with GeneMapper Software version 4.0 (Life Technologies). STR typing followed the manufacturer’s protocol using provided locus panels and allele bins, with allele designations matching the supplied allelic ladder. Genotype nomenclature followed recommendations from the International Society for Forensic Genetics (ISFG) [23,27].

### 2.4. Statistical Analyses

Observed heterozygosity (HO), expected heterozygosity (HE), and Hardy–Weinberg equilibrium (HWE) for female samples, as well as linkage disequilibrium (LD) for all loci pairs (male and female), were calculated using Arlequin v3.5 [28]. Allele frequencies were determined by counting occurrences of each allele and expressing them as fractions of the total. Fisher’s exact test was performed using an online tool (https://www.socscistatistics.com, accessed on 1 September 2024). Haplotype frequencies for four linkage groups (LG1, LG2, LG3, and LG4) [29,30] were calculated by counting observed haplotypes and computing haplotype diversity (HD) using the formula mentioned in Equation (1):

(1) $$HD=\frac{n}{n-1}\left(1-\sum_{i}p_{i}^{2}\right)$$

where *n* is the male population size and *pi* is the frequency of the *i*th haplotype. Other forensic statistical parameters, including the power of discrimination in females (PDF) and males (PDM), polymorphism information content (PIC), power of exclusion (PE), and various mean paternity exclusion chances (MECKrüger, MECKishida, MECDesmarais, and MECDesmarais Duo variants), were estimated using the online tool provided by the ChrX-STR.org 2.0 database [31]. Nei’s genetic distances between Punjabi, Kashmiri, and seventeen reference populations [19,25,32,33,34,35,36,37,38] were calculated with Phylip 3.695 software [39], and a neighbor-joining tree was constructed using Mega 7.0 [40]. Principal component analysis (PCA) based on allele frequency correlation was conducted by using the MVSP v3.22 software (http://www.kovcomp.com). The STRUCTURE v.2.3.4 software [41] was used to calculate the ancestry component. The model-based analysis employed the length of the burnin period of 100,000 and Markov Chain Monte Carlo (MCMC) step of 100,000 under the ‘independent allele frequencies’ and ‘LOCPRIOR’ models with the k values ranging from 2 to 10 with 5 repeats per run.

## 3. Results and Discussion

### 3.1. Forensic Parameter Analysis

The allele frequencies of Punjabi and Kashmiri populations are summarized in Table S1. In the Kashmiri population, no significant difference was found between male and female allele frequencies (*p* > 0.05) by the Exact Test; therefore, males and females were pooled for calculating forensic parameters. The most polymorphic locus was DXS10134 and DXS10148 with 20 alleles, while DXS7132 was the least polymorphic locus with 8 alleles, and a total of 166 unique alleles were observed with frequencies ranging from 0.0066 to 0.4266. In the Punjabi population, DXS10148 was the most polymorphic locus with 25 allelic combinations, while DXS7132 was the least polymorphic locus with 8 different allelic combinations, and a total of 171 unique alleles were observed with frequencies ranging from 0.0102 to 0.3367. In the Kashmiri population initially, seven loci were not in Hardy–Weinberg Equilibrium (HWE) in the females. Nevertheless, when a sequential Bonferroni [42] adjustment was used to avoid the so-called “multiple comparison problem” (where with a significance threshold *p*-value of 0.05, 5% of tests are likely to be significant by chance), only two loci (HPRTB and DXS10148) were found to be out of HWE (Table S2). The same trend was observed in the Punjabi population, where initially nine loci were out of HWE. After applying the sequential Bonferroni correction [42], only three loci (DXS10079, DXS10103, and DXS10134) were out of HWE. In the Punjabi population, the X-STR marker DXS10148 had the highest forensic utility with a PIC (Polymorphic Information Content) value of 0.9377, while DXS8378 had the lowest PIC value of 0.7288. In the Kashmiri population, DXS10135 had the highest PIC value at 0.9267, and DXS7423 had the lowest at 0.6938. The combined power of Exclusion (CPE) across 12 X STRs was 0.999 999 92445 in Punjabi and 0.999 999 6546 in the Kashmiri population. The combined power of discrimination (including all 12 X-STR loci) was 0.999 999 998 4256 for males and 0.999 999 999 999 999 997 574 for females, while mean exclusion chances (MECKrüger, MECKishida, MECDesmarais, and MECDesmarais Duo) were 0.999 999 942, 0.999 999 999 949, 0.999 999 999 949, and 0.999 999 97768, respectively in Punjabi. The combined power of discrimination (CPD) in Kashmiri was 0.999 999 999 92276 for males and 0.999 999 999 999 999 995644 for females, and mean exclusion chances (MECKrüger, MECKishida, MECDesmarais, and MECDesmarais Duo) were 0.999 999 7561, 0.999 999 9997301, 0.999 999 9997318, and 0.999 999 90897, respectively (Table 1).

These findings indicate that the twelve X-STR loci are useful for forensic identification and paternity testing in both the Punjabi and Kashmiri populations residing in the Punjab and Azad Kashmir regions of Pakistan. 

### 3.2. Linkage Disequilibrium Analyses

In a population undergoing random mating with no selection, migration, or mutation, loci will approach LE over time [43]. When in LE, alleles are expected to be randomly assorted, and the allele frequency at one locus is independent of the allele frequency at another locus. However, selective pressures, population structure, or low recombination rates will favor LD, particularly when loci are physically close to each other on the same chromosome. When in LD, alleles at different loci are inherited together more often than would be expected by chance. Understanding the balance between these states informs about both evolutionary history and current genetic diversity in populations. Exact tests for LE showed that the *p*-values of 32 pairwise combinations of STR loci were below 0.05 and thus displayed LD in Kashmiri females (Table S3). After applying the sequential Bonferroni correction [30] only twenty pairs (DXS10146/HPRTB, DXS10135/DXS8378, DXS10079/DXS7423, DXS10146/DXS7423, DXS10148/DXS7423, DXS10079/DXS10148, DXS10103/DXS10148, DXS10134/DXS10148, DXS10146/DXS10148, DXS7423/DXS10148, DXS10148/DXS10146, DXS7423/DXS10146, HPRTB/DXS10146, DXS8378/DXS10135, DXS10148/DXS10134, DXS10079/DXS10103, DXS10148/DXS10103, DXS10103/DXS10079, DXS10148/DXS10079, and DXS7423/DXS10079) were still showing LD. In the Punjabi female population, exact tests for LE showed that the *p*-values of 44 pairwise combinations of STR loci were below 0.05, and thus displaying LD. After applying the sequential Bonferroni correction [30], only fourteen pairs (DXS10101/DXS10146, DXS10146/DXS10101, DXS10146/HPRTB, DXS10101/DXS7132, DXS10079/DXS10148, DXS10146/DXS10148, DXS10135/DXS10146, DXS10148/DXS10146, HPRTB/DXS10146, DXS10146/DXS10135, DXS10101/DXS10134, DXS10134/DXS10101, DXS7132/DXS10101, and DXS10148/DXS10079) were still out of LE (Table S3). LD can occur when alleles that are close to each other on the same chromosome are passed down together more often than would be expected by chance, typically due to inheritance from a common ancestor. As mentioned before, LD can occur due to low recombination rates, when alleles are very close to each other on the same chromosome and are thus inherited together more often than expected by chance, typically due to inheritance from a common ancestor. However, LD can also be favored by natural selection, random changes in gene frequency (genetic drift), differences in mutation or recombination rates, mating that isn’t random, effects from a small initial population (founder effects), biases in gene sampling, mixing of different populations (recent admixture), and the existence of distinct groups within a population (population substructure) [44].

### 3.3. Haplotype and LD Analysis in Kashmiri Males

The 12 X-STR markers are clustered into four linkage groups based on their physical localizations [29,30]: LG1 (Xp22) contains DXS8378-DXS10135-DXS10148, LG2 (Xq11) contains DXS7132-DXS10074-DXS10079, LG3 (Xq26) contains DXS10101-DXS10103-HPRTB, and LG4 (Xq28) contains DXS7423-DXS10134-DXS10146. Each cluster of three markers is considered as one haplotype for the genotyping of males. For these four linkage groups (LG1, LG2, LG3, and LG4), the numbers of observed haplotypes in Kashmiri males were 34, 31, 28, and 32, respectively, while haplotype diversities were 0.9697, 0.9677, 0.9642, and 0.9687, respectively (Table S4). The number and frequencies of the most common haplotypes in the four linkage groups were as follows: 2 males with a frequency of 0.0571 (LG1); 2 males with a frequency of 0.0571 (LG2); 3 males with a frequency of 0.0857 (LG3); and 2 males with a frequency of 0.0571 (LG4). If the STR trios within the four linkage groups formed stable haplotypes, we would anticipate strong linkage among them while expecting no linkage between STRs in different linkage groups. However, as shown in Table S4, these expectations were not fulfilled. Only DXS10135/DXS10148 in LG1, DXS10101/DXS10103 in LG3, DXS10146/DXS7423, and DXS10134/DXS7423 in LG4 show significant LD within linkage groups before applying the sequential Bonferroni correction. When sequential Bonferroni correction was applied, none of the pairs showed any LD. We also observed significant LD between the following groups: DXS10079(LG2)-DXS8378(LG1); DXS10101(LG3)-DXS7132(LG2); and DXS7132(LG2)-DXS8378(LG-1) before applying the sequential Bonferroni correction. When sequential Bonferroni correction was applied, none of the pairs showed any LD. Linkage expectations were solely based on the physical distances between loci; however, LD can also arise from factors such as random genetic drift, founder effects, mutations, selection, and population admixture or stratification [45,46]. Several studies have shown that loci within each of the four X-STR linkage groups exhibit low but non-zero recombination rates [47]. Furthermore, recombination between these linkage groups is less than 50% [30,48,49]. Consequently, Diegoli et al. [30] have argued against the concept of independent X chromosomal linkage groups due to these factors.

### 3.4. Inter-Population Differentiation

**Figure 2:**
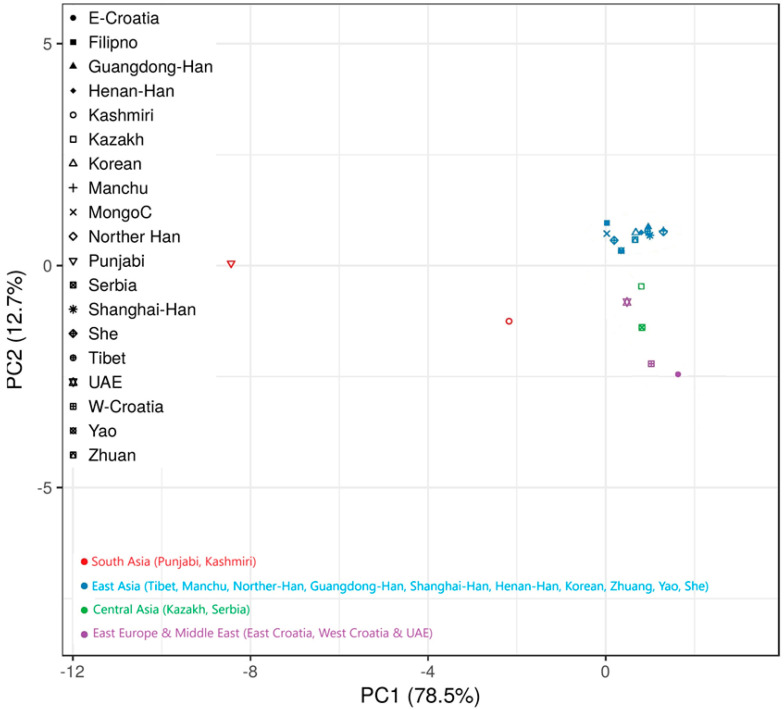
Principal component analysis (PCA) based on Nei’s genetic distance revealed by the first two components between the Kashmiri and Punjabi populations from Pakistan and other worldwide populations.

To check the existence of population genetic differences in Kashmiri and Punjabi with other reference populations with raw genotypic data, we check the genetic homozygosity or heterozygosity via principal component analysis (PCA). On the *x*-axis, representing 78.5% of the variance, PC1 separates populations mostly along an East-West geographic axis. The large percentage of variation explained by PC1 suggests that the most significant genetic variation among these populations is related to this gradient. The *y*-axis represents Principal Component 2 (PC2), which accounts for 12.7% of the variance. PC2 likely captures genetic diversity that is not associated with the East-West gradient. This could correspond to a North-South divide or another genetic gradient (Figure 2).

The Punjabi population is positioned on the left side of the plot, indicating significant genetic differentiation from the other Asian populations. The Kashmiri population is also positioned on the left, but slightly below the Punjabi population on the PC2 axis, showing some differentiation between these two populations as well. Populations from East Asia (like the Han Chinese subgroups and Korean) are clustered together towards the right of the plot, separated from the South Asian groups by PC1. Populations from Central Asia (like Kazakh and Mongol) and Eastern Europe (like Croatians) are spread out between the South Asian and East Asian clusters, suggesting intermediate genetic variation relative to these regions. Punjabi and Kashmiri populations show clear genetic distinction from East Asian populations. The spread along PC1 indicates that these South Asian populations have unique genetic variations that set them apart from East Asian and other populations included in this analysis. The separation of Punjabi and Kashmiri along PC2, although representing a smaller proportion of the genetic variance, suggests that there are genetic differences between these two populations as well, though they are much less pronounced than the differences between South Asian and East Asian groups.

**Figure 3:**
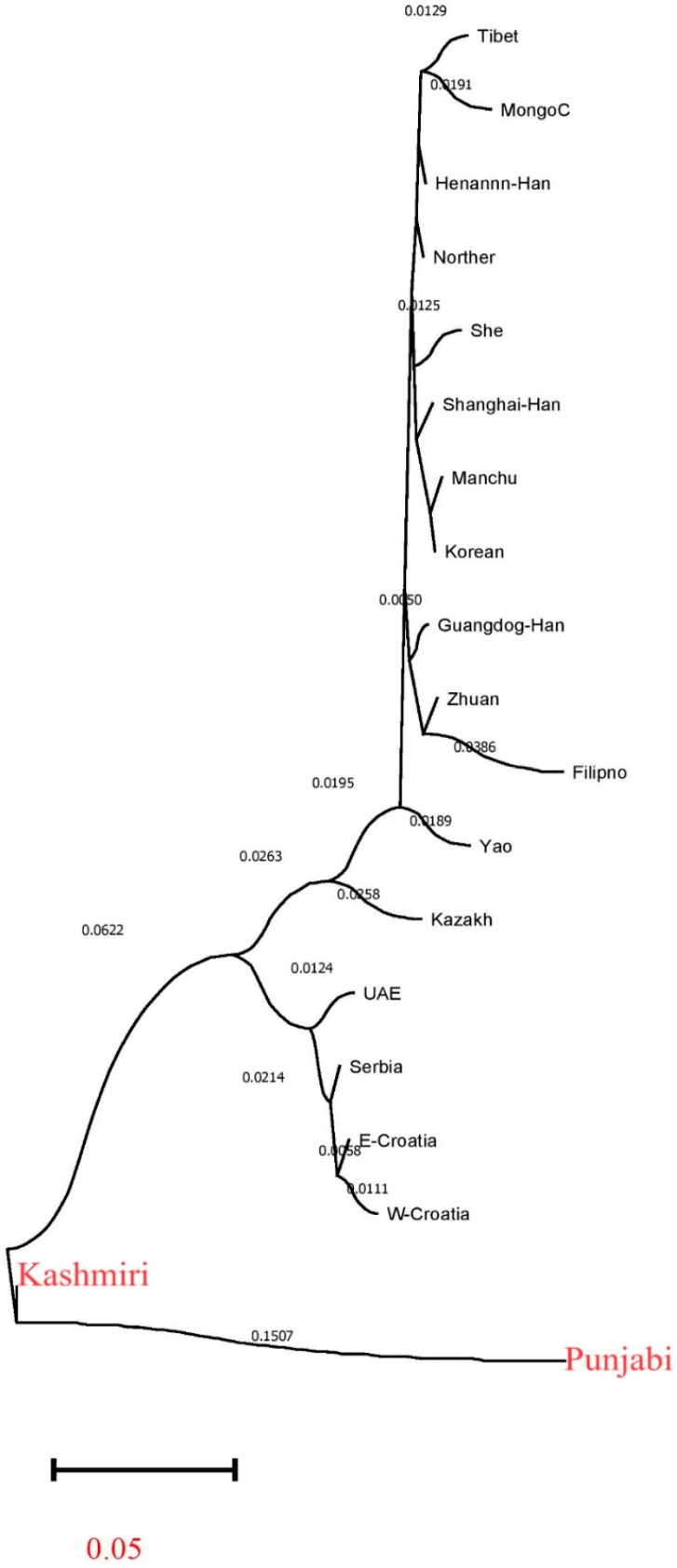
Neighbor-joining tree of the Punjabi and Kashmiri populations from Pakistan in relation to other populations.

Then we explored the genetic affinities between Kashmiri, Punjabi, and 17 reference populations. Nei’s genetic distances between Kashmiri and 18 reference worldwide populations are summarized in Table S5. The Neighbor-Joining (NJ) tree represents genetic distances, illustrating the evolutionary paths of the studied populations and highlighting their different levels of divergence from a shared ancestral lineage [50]. The Kashmiri population’s extended branch length on the NJ tree signifies a pronounced genetic departure, potentially attributable to a confluence of genetic drift, historical isolation, or selective forces. Conversely, the Punjabi population’s relatively shorter branch suggests a lesser degree of genetic separation, indicative of a more recent common ancestry or ongoing gene flow with the rest of the populations represented (Figure 3). The choice of X-chromosome Short Tandem Repeats (X-STRs) is crucial due to their polymorphic nature and high mutation rates, making them especially useful for assessing genetic diversity within populations. These markers provide a lattice upon which the NJ tree’s architecture is constructed, with their mutation rates intimately linked to the patterning of genetic distances observed. These high mutation rates likely enable a clear distinction of the populations on the tree, particularly highlighting the divergent branch of the Punjabi population.

**Figure 4:**
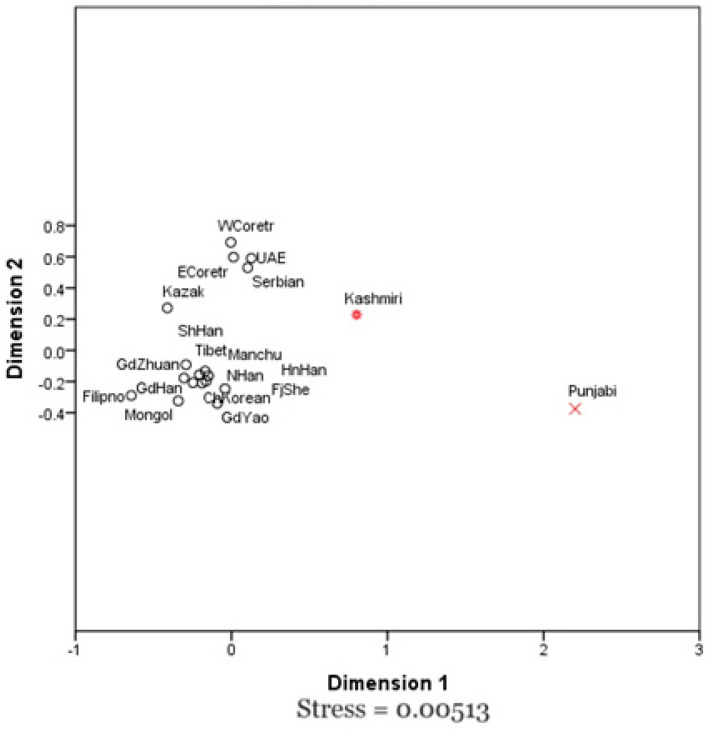
A two-dimensional scaling (MDS) plot of the Kashmiri and Punjabi populations from Pakistan in relation to other populations based on Nei’s genetic distance (* & × are representing currently studied populations).

The Multi-Dimensional Scaling (MDS) plot presents a two-dimensional map of genetic distances among the populations, derived from Nei’s genetic distance measurements. While Principal Component Analysis (PCA) seeks to explain the maximum variance using the primary components, MDS focuses on maintaining the relative distances between populations, giving a complementary perspective on genetic relationships. In Figure 4, the Punjabi and Kashmiri populations are distinctly marked, showing clear separation from the other groups, particularly from East Asian and Central Asian populations. The horizontal axis (Dimension 1) seems to capture the bulk of genetic differences, which may correspond to historical and geographic factors that shaped migration patterns and genetic drift. Meanwhile, the vertical axis (Dimension 2) accounts for more subtle genetic variations, which could be due to regional factors such as cultural isolation or practices like endogamy.

The Punjabi population, located far along Dimension 1, displays significant genetic divergence from both East and Central Asian groups. This could suggest a long-term genetic isolation of Punjabis, possibly due to restricted gene flow with neighboring populations, leading to a more distinct genetic profile. This isolated positioning aligns with the results from both the PCA and the Neighbor-Joining (NJ) tree, reinforcing the unique nature of the Punjabi genetic makeup.

Conversely, the Kashmiri population is positioned closer to Central and East Asian groups, indicating moderate genetic differentiation. This placement may reflect historical gene flow through trade or migration routes that brought new genetic influences. Additionally, the shorter genetic distance between Kashmiris and certain East Asian populations hints at shared ancestry or possible admixture events. This observation aligns with other analyses, such as the heatmap and NJ tree, where Kashmiris show a mix of unique and shared genetic traits with both South Asian and East Asian groups.

The MDS plot reveals a larger genetic gap between the Punjabi and Kashmiri populations compared to the more minimal differences observed among East Asian populations. Despite their genetic distinctions, Punjabi and Kashmiri groups share a closer relationship with each other than with populations located further east or west. The outlier status of the Punjabi population in the MDS plot may reflect stronger evolutionary forces or selective pressures acting on this group, contributing to their greater genetic divergence. The stress value of 0.00513 in the MDS plot is quite low, indicating that the two-dimensional layout accurately preserves the genetic distances between populations with little distortion. This suggests that the relationships shown in the plot reliably represent the true genetic connections among the studied groups.

**Figure 5:**
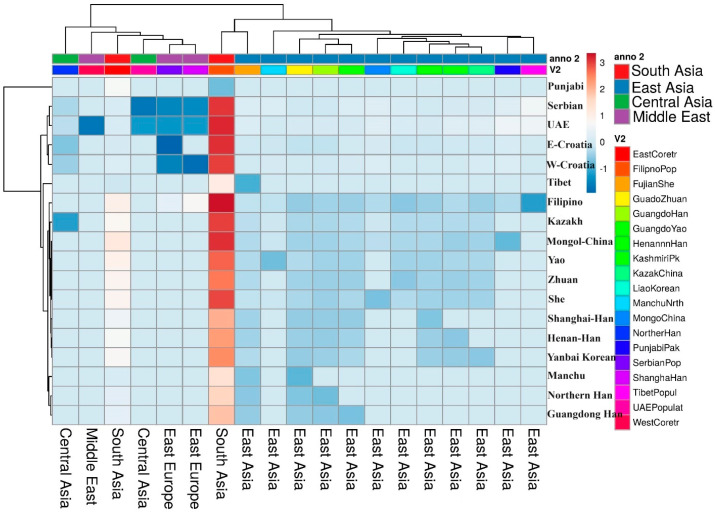
A heat map of pairwise Nei’s genetic distance values between the Kashmiri and Punjabi populations from Pakistan in relation to other worldwide populations.

The results from the MDS plot align closely with those of the NJ tree, highlighting the interplay of various population genetic forces. Genetic drift introduces random fluctuations in allele frequencies, while migration brings in new genetic material, both of which are critical drivers of genetic differentiation. Additionally, societal practices such as endogamy [51,52,53,54], which restricts outbreeding, further shape the unique genetic structures within these populations. When placed in a comparative context, the NJ tree underscores the distinctiveness of the Punjabi and Kashmiri populations in relation to their Asian counterparts. This distinct positioning suggests a complex genetic history influenced by numerous demographic events, including migrations, admixtures, and social interactions that have occurred over time. The heat map of the genetic matrix reveals a unique pattern for the Punjabi population, marked by high expression (indicated by red color) for certain genetic markers that are also somewhat prominent in the Kashmiri population, as well as in populations from East Croatia and the UAE (Figure 5) [55]. This similarity might suggest a shared genetic background or common genetic traits among these groups, potentially influenced by historical trade routes, cultural exchanges, and intermarriages. Conversely, while the Kashmiri population exhibits some genetic similarity to the Punjabi population [3], it also showcases unique characteristics, as evidenced by the distinct color patterns in the heatmap. This implies that, despite some overlap in their genetic makeup, significant differences still exist that distinguish the Kashmiri population. The clustering observed in the top dendrogram indicates that the Punjabi and Kashmiri populations are closely related to one another and to other South Asian populations, yet remain distinct from East Asian and Central Asian populations, as illustrated by the longer branches separating these groups. Additionally, the clustering on the left dendrogram suggests that certain genetic markers are more prevalent in South Asian populations, including the Punjabis and Kashmiris, than in other regions. This pattern may reflect specific evolutionary pressures, historical migrations, and socio-cultural interactions that have shaped the genetic landscape of South Asia.

### 3.5. Ancestry Content Analysis with Structure

**Figure 6:**
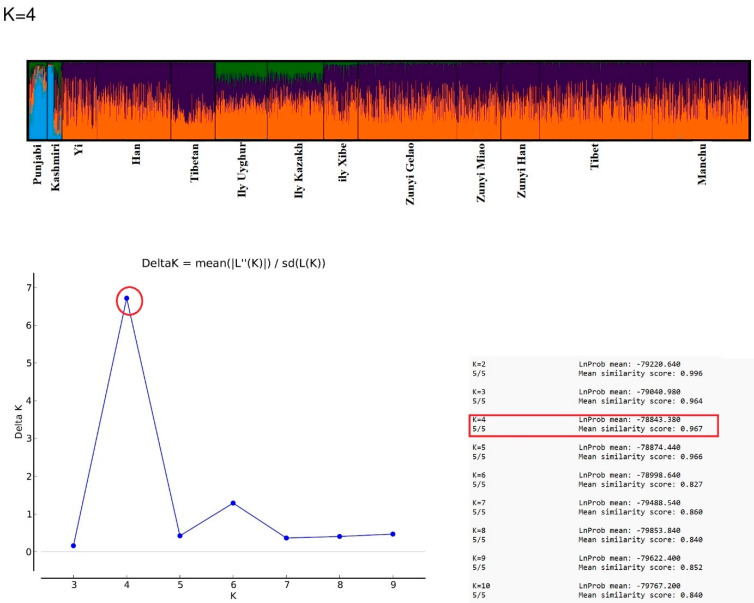
Ancestry content analysis with STRUCTURE.

The STRUCTURE analysis conducted on the Kashmiri and Punjabi populations, alongside 11 Chinese populations from China, reveals significant insights into the genetic diversity and ancestral composition of these groups. By employing a model-based clustering algorithm to explore ancestry content, this study identified the optimal number of ancestral populations as four (K = 4), with a high mean similarity score of 0.967, suggesting a robust model fit (Figure 6).

The STRUCTURE analysis at K = 4 elucidates the intricate tapestry of ancestral genetic components within the sampled populations, differentiated by four distinct colors: blue, orange, purple, and green. These colors demarcate the proportionate ancestry each shares with the four inferred genetic clusters. The analysis reveals that for the Kashmiri and Punjabi populations, a predominant blue component characterizes their genetic structure, pointing to a shared major ancestral background. Notably, traces of green, purple, and orange within their genetic profiles suggest historical admixture with the three other ancestral groups identified, possibly reflecting past migrations, social interactions, or invasions that have introduced genetic diversity. The genetic distinctions between the Punjabi and Kashmiri populations stem from a multifaceted interaction of historical, linguistic, and socio-cultural elements. Punjab’s strategic position as a nexus for migrations, beginning with the arrival of the Aryans around 1500 BCE and followed by invasions from groups such as the Indo-Greeks, Kushans, and Huns, has significantly influenced its genetic makeup [56]. The region’s integration into powerful empires, including the Maurya, Gupta, and Mughal, further enhanced genetic and cultural exchanges through trade and conquest. In contrast, Kashmir’s geographical seclusion has limited extensive migrations, although its genetic diversity has still been shaped by influences from Persian, Turkic, and Islamic conquests. The region has also been home to its own dynasties, like the Karkota and Utpala, which contributed to maintaining distinct genetic traits. However, despite Kashmir’s relative geographical isolation, genetic data, including the NJ tree, MDS plot, and heatmap, show that the Kashmiri population is genetically closer to certain populations than previously thought. This proximity reflects selective historical migrations and gene flow, particularly through trade routes and conquests, even though it has retained unique genetic traits due to localized isolation. In contrast, the Punjabi population’s greater genetic divergence from neighboring populations, as evidenced in these analyses, aligns with its history as a genetic melting pot. The extensive gene flow into Punjab from its position as a hub for migration and invasion has made it more genetically diverse, which is evident from its distinct positioning on the MDS plot and NJ tree.

From a linguistic perspective, Punjabi serves as a crucial marker of the Indo-Aryan heritage in Punjab, deeply intertwined with the local culture. In Kashmir, the languages spoken—Kashmiri, Urdu, and Dogri—reflect a wider historical tapestry that includes Persian and Arabic influences. These linguistic variations have helped shape the cultural identities of both groups, affecting societal norms and marriage practices. Endogamy, in particular, has played a vital role in both populations, reinforcing genetic homogeneity within communities [1]. While Punjab’s extensive trade routes facilitated a greater exchange of genetic material, Kashmir’s relative isolation and selective trade interactions helped preserve certain unique genetic traits.

Moreover, socio-political events, particularly the Partition of India in 1947, have had profound effects on the genetic structure of the Punjabi population, resulting in significant migrations. Ongoing political tensions in Kashmir have similarly impacted the genetic dynamics of its population, leading to a more insular genetic profile [57]. This distinction is captured in genetic data, which shows that while the Kashmiri population shares some genetic traits with neighboring populations due to historical migrations, it also maintains a distinct genetic structure compared to the more genetically diverse Punjabi population.

A thorough understanding of the genetic variations between Punjabi and Kashmiri groups necessitates an exploration of their historical, linguistic, and socio-cultural backgrounds. The convergence of historical migrations, linguistic diversity, cultural practices, and socio-political influences has shaped the genetic diversity seen in these populations. By examining these factors, we can attain a deeper understanding of the evolutionary processes that inform the genetic landscape of South Asia.

In stark contrast, the majority of the other studied populations, likely representing diverse regions within China, are characterized predominantly by two genetic components, orange and purple. This binary composition may indicate a more homogeneous population history or a stable population structure with minimal admixture or external genetic influences. Standing out from this pattern, the Kazakh and Uyghur populations exhibit a notable presence of the green component along with the orange and purple, hinting at a distinct genetic influence that is not as prevalent in the other populations. This may suggest a unique ancestral background or more recent gene flow from neighboring populations not encompassed within the scope of this analysis.

## 4. Conclusions

This study provides a comprehensive analysis of the genetic landscape of the Punjabi and Kashmiri populations in Pakistan, with a focus on X-STR loci. The forensic parameter analysis demonstrates the high polymorphism and discriminative power of the 12 X-STR loci examined, particularly in the DXS10148 locus, across both populations. The results affirm the utility of these loci in forensic applications, including identity verification and paternity testing, within these populations. LD analyses revealed significant LD among several X-STR loci within and between linkage groups, suggesting complex genetic interactions and possible evolutionary forces at play, such as genetic drift, population substructure, and admixture events. However, after correcting for multiple comparisons, the observed LD was largely reduced, indicating that most of the X-STR loci assort independently in these populations.

Haplotype analysis in Kashmiri males further elucidated the genetic diversity within linkage groups, showing high haplotype diversity, although expectations of tight linkage within groups were not consistently met. The inter-population differentiation analysis highlighted the genetic distinctiveness of Punjabi and Kashmiri populations from East Asian populations, as well as subtle genetic differences between the two South Asian groups themselves. These differences are aligned with historical and geographical influences on these populations, underscored by PCA and Nei’s genetic distance analyses, which suggest a complex demographic history shaped by migration, isolation, and admixture. The STRUCTURE analysis reinforced these findings by revealing a predominant ancestral component shared between the Punjabi and Kashmiri populations, alongside evidence of historical admixture with other genetic groups. This contrasts with the more homogeneous genetic profiles observed in East Asian populations, indicating a distinct and diverse genetic heritage within the studied South Asian populations.

In summary, this study provides valuable insights into the genetic structure of Punjabi and Kashmiri populations, highlighting their forensic relevance and the broader implications of their genetic diversity in understanding population history and dynamics in South Asia. X-STRs have several advantages in forensic applications, particularly in scenarios involving kinship analysis, where the inheritance patterns of X-linked markers can provide critical insights. However, their application is not without challenges. One significant limitation is that X-STRs often exhibit reduced discriminatory power compared to autosomal markers [58]. This reduced power is particularly evident in complex familial relationships or when analyzing populations with limited genetic diversity, where autosomal markers can provide more robust differentiation. Furthermore, the presence of recombination events during meiosis can complicate the interpretation of X-STR data over generations. Recombination may lead to the loss of LD between X-STRs and nearby genetic markers, resulting in challenges when estimating allele frequencies or inferring ancestry. Additionally, the potential for homoplasy where similar alleles arise independently rather than from a common ancestor can further reduce the reliability of X-STRs in some forensic contexts. Future studies should aim to expand these findings by including a broader range of populations and genetic markers to further unravel the complex genetic tapestry of this region.

## Figures and Tables

**Table 1:** Forensic parameters of the 12 X-STR loci in the Punjabi and Kashmiri population from Pakistan.

**Punjabi Population**												
**General**	**DXS10074**	**DXS10079**	**DXS10101**	**DXS10103**	**DXS10134**	**DXS10135**	**DXS10146**	**DXS10148**	**DXS7132**	**DXS7423**	**DXS8378**	**HPRTB**
Polymorphism information content (PIC):	0.850481	0.85732	0.91789	0.77079	0.92401	0.91509	0.87054	0.93771	0.76708	0.80966	0.72889	0.79844
Homozygotie (h):	0.135368	0.12974	0.07684	0.20179	0.07143	0.07934	0.11891	0.05914	0.20304	0.16951	0.23532	0.17909
Heterozygotie (HET):	0.864632	0.87026	0.92316	0.79821	0.92857	0.92066	0.88109	0.94086	0.79696	0.83049	0.76468	0.82091
Power of Exclusion (PE):	0.723898	0.73517	0.84293	0.59572	0.85407	0.83779	0.75698	0.87939	0.59341	0.65679	0.53522	0.6384
**Power of Discrimination**												
PD female:	0.967524	0.97023	0.98883	0.93186	0.99034	0.98814	0.97531	0.99335	0.92889	0.95044	0.90884	0.94545
PD male:	0.864632	0.87026	0.92316	0.79821	0.92857	0.92066	0.88109	0.94086	0.79696	0.83049	0.76468	0.82091
**Mean paternity exclusion change**												
MEC Krüger:	0.730323	0.74193	0.84393	0.61043	0.85516	0.839	0.76498	0.88039	0.60154	0.6664	0.55161	0.65079
MEC Kishida:	0.850424	0.85732	0.91789	0.77079	0.92401	0.91509	0.87054	0.93771	0.76707	0.80966	0.72889	0.79843
MEC Desmarais:	0.850481	0.85732	0.91789	0.77079	0.92401	0.91509	0.87054	0.93771	0.76708	0.80966	0.72889	0.79844
MEC Desmarais Duo:	0.751926	0.76179	0.85309	0.64618	0.86315	0.84862	0.78139	0.88602	0.64098	0.69592	0.59502	0.6816
**Kashmiri Population**												
**General**	**DXS10074**	**DXS10079**	**DXS10101**	**DXS10103**	**DXS10134**	**DXS10135**	**DXS10146**	**DXS10148**	**DXS7132**	**DXS7423**	**DXS8378**	**HPRTB**
Polymorphism information content (PIC):	0.838077	0.841783	0.90057	0.816471	0.896887	0.9267	0.845954	0.919169	0.726672	0.693819	0.705525	0.75258
Homozygotie (h):	0.146314	0.143381	0.092223	0.164625	0.095524	0.069067	0.138891	0.076001	0.238046	0.270047	0.252458	0.214579
Heterozygotie (HET):	0.853686	0.856619	0.907777	0.835375	0.904476	0.930933	0.861109	0.923999	0.761954	0.729953	0.747542	0.785421
Power of Exclusion (PE):	0.702143	0.707951	0.811335	0.666253	0.804572	0.858939	0.716874	0.844661	0.530438	0.476104	0.505569	0.572268
**Power of Discrimination**												
PD female:	0.962983	0.964606	0.984287	0.953994	0.983286	0.990996	0.965554	0.989394	0.908052	0.89094	0.894248	0.921115
PD male:	0.853686	0.856619	0.907777	0.835375	0.904476	0.930933	0.861109	0.923999	0.761954	0.729953	0.747542	0.785421
**Mean paternity exclusion change**												
MEC Krüger:	0.711853	0.717928	0.813577	0.679084	0.807815	0.860229	0.722474	0.847645	0.549182	0.51467	0.519001	0.580394
MEC Kishida:	0.838077	0.841783	0.900245	0.816471	0.896887	0.9267	0.845619	0.919169	0.726672	0.693819	0.705289	0.75258
MEC Desmarais:	0.838077	0.841783	0.90057	0.816471	0.896887	0.9267	0.845954	0.919169	0.726672	0.693819	0.705525	0.75258
MEC Desmarais Duo:	0.734901	0.740012	0.825955	0.705674	0.820385	0.867655	0.745607	0.855874	0.592169	0.554842	0.567886	0.622908

## Data Availability

All data generated or analyzed during this study are included in this article and its Supplementary Information Files.

## Supplementary Materials

The following supporting information can be downloaded at: https://www.mdpi.com/article/10.3390/genes15111384/s1, Table S1: Allele Frequencies; Table S2: HWE; Table S3: LE; Table S4: hd, LD among LG1–4 groups; Table S5: Nei’s genetic distance.
